# Hoechst-tagged Fluorescein Diacetate for the Fluorescence Imaging-based Assessment of Stomatal Dynamics in *Arabidopsis thaliana*

**DOI:** 10.1038/s41598-020-62239-w

**Published:** 2020-03-24

**Authors:** Yousuke Takaoka, Saki Miyagawa, Akinobu Nakamura, Syusuke Egoshi, Shinya Tsukiji, Minoru Ueda

**Affiliations:** 1 Department of Chemistry, Graduate School of Science, Tohoku University, Sendai, 980-8578 Japan; 2 Precursory Research for Embryonic Science and Technology (PREST), Japan Science and Technology Agency, 5 Sanbancho, Chiyoda-ku, Tokyo, 102-0075 Japan; 3 Department of Molecular and Chemical Life Sciences, Graduate School of Life Sciences, Tohoku University, Sendai, 980-8578 Japan; 4 Department of Life Science and Applied Chemistry, Nagoya Institute of Technology, Gokiso-cho, Showa-ku, Nagoya, 466-8555 Japan; 5 Department of Nanopharmaceutical Sciences, Nagoya Institute of Technology, Gokiso-cho, Showa-ku, Nagoya, 466-8555 Japan; 6 Frontier Research Institute for Materials Science (FRIMS), Nagoya Institute of Technology, Gokiso-cho, Showa-ku, Nagoya, 466-8555 Japan

**Keywords:** Chemical tools, Fluorescent probes

## Abstract

In plants, stomata regulate water loss through transpiration for plant growth and survival in response to various environmental stressors; and simple methods to assess stomatal dynamics are needed for physiological studies. Herein, we report a fluorescence-imaging-based method using fluorescein diacetate tagged with Hoechst 33342, a nuclear staining chemical probe (HoeAc_2_Fl) for the qualitative assessment of stomatal dynamics. In our method, the stomatal movement is inferred by simple monitoring of the fluorescence intensity in the nucleus of the stomata.

## Introduction

Stomatal dynamics influence plant transpiration, gas exchange, drought tolerance, and defense^1,2^; and stomatal movement takes place in response to several environmental stimuli, such as blue light, red light, low CO_2_, and chemicals. The signaling mechanisms that underly stomatal movement have attracted the attention of plant physiologists^3–5^, and there has been a great deal of research interest into the development of chemical tools for the study of stomatal movements^6–12^. For example, in pioneering work, Cutler *et al*. reported pyrabactin, a synthetic agonist of abscisic acid (ABA) receptors, which led to their identification and the development of stomatal closing agents^6,13^; and Kinoshita *et al*. identified several small molecules that affect stomatal movements from a chemical library^9^. However, to speed the development of molecules capable of affecting stomatal movements, simple and high-throughput methods for chemical screening are urgently needed. Stomatal movements are currently evaluated by either the direct monitoring of stomatal aperture under the microscope, or analyses of thermal images, that reflect the degree of transpiration from stomata^14–16^. Although these methods are robust and reliable, the former is time-consuming and low throughput, the latter requires the special equipment^17^.

**Figure 1:**
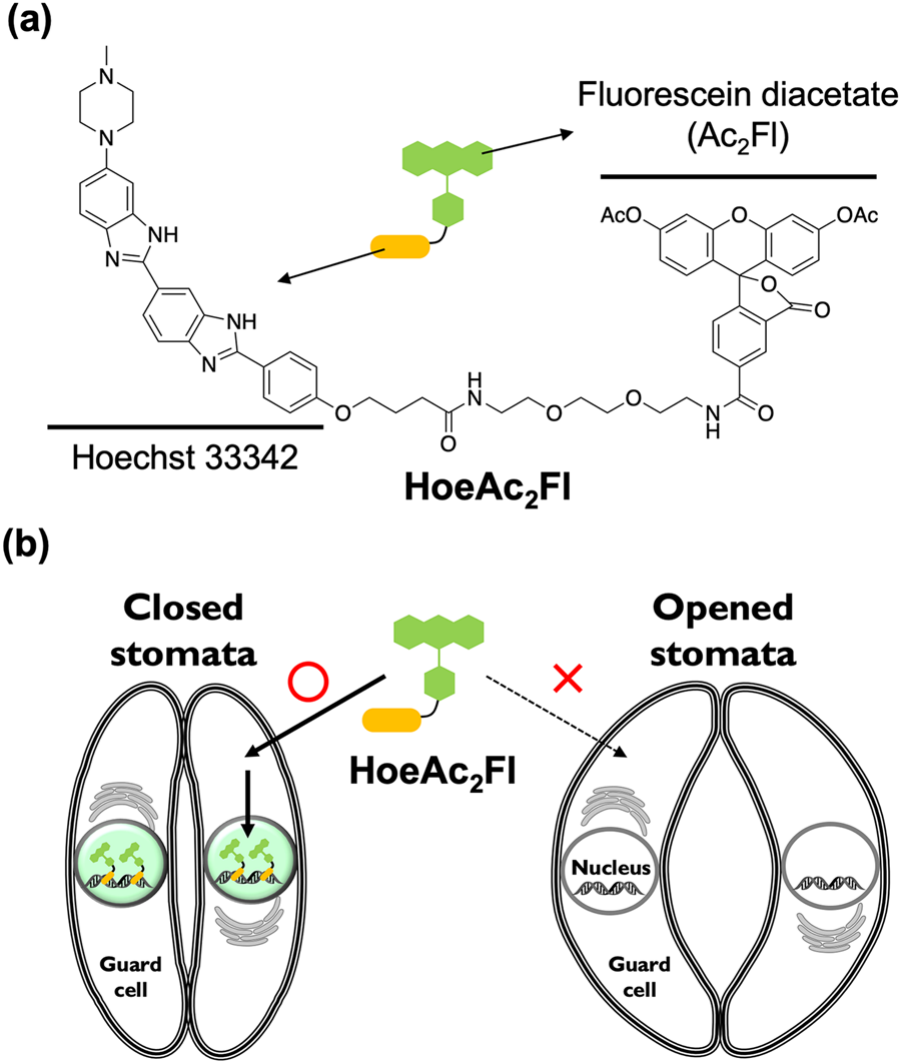
(**a**) Chemical structure of HoeAc_2_Fl which comprises Hoechst 33342 and fluorescein diacetate moieties. (**b**) Schematic illustration of selective staining of the nucleus in the closed stomata by HoeAc_2_Fl.

Herein, we report a simple and convenient method for the assessment of stomatal closing/opening in *Arabidopsis thaliana* based on fluorescent live imaging by Hoechst-tagged acetylfluorescein (HoeAc_2_Fl, Fig. 1a). Our method enables objective assessment of stomatal dynamics by simple monitoring of the fluorescence intensity of HoeAc_2_Fl in the nucleus of the stomata.

## Results and Discussion

HoeAc_2_Fl is a fluorescent stain comprising Hoechst 33342 and fluorescein diacetate moieties, and was originally developed for nuclear staining of mammalian cells^18,19^. We discovered the guard cells of closed stomata can be selectively stained by application of HoeAc_2_Fl to the plant (Fig. 1b). Therefore, we proposed that HoeAc_2_Fl could constitute a useful chemical tool for the assessment of stomatal dynamics by simple monitoring of the intensity of the fluorescence of the nuclei of the guard cells.

**Figure 2:**
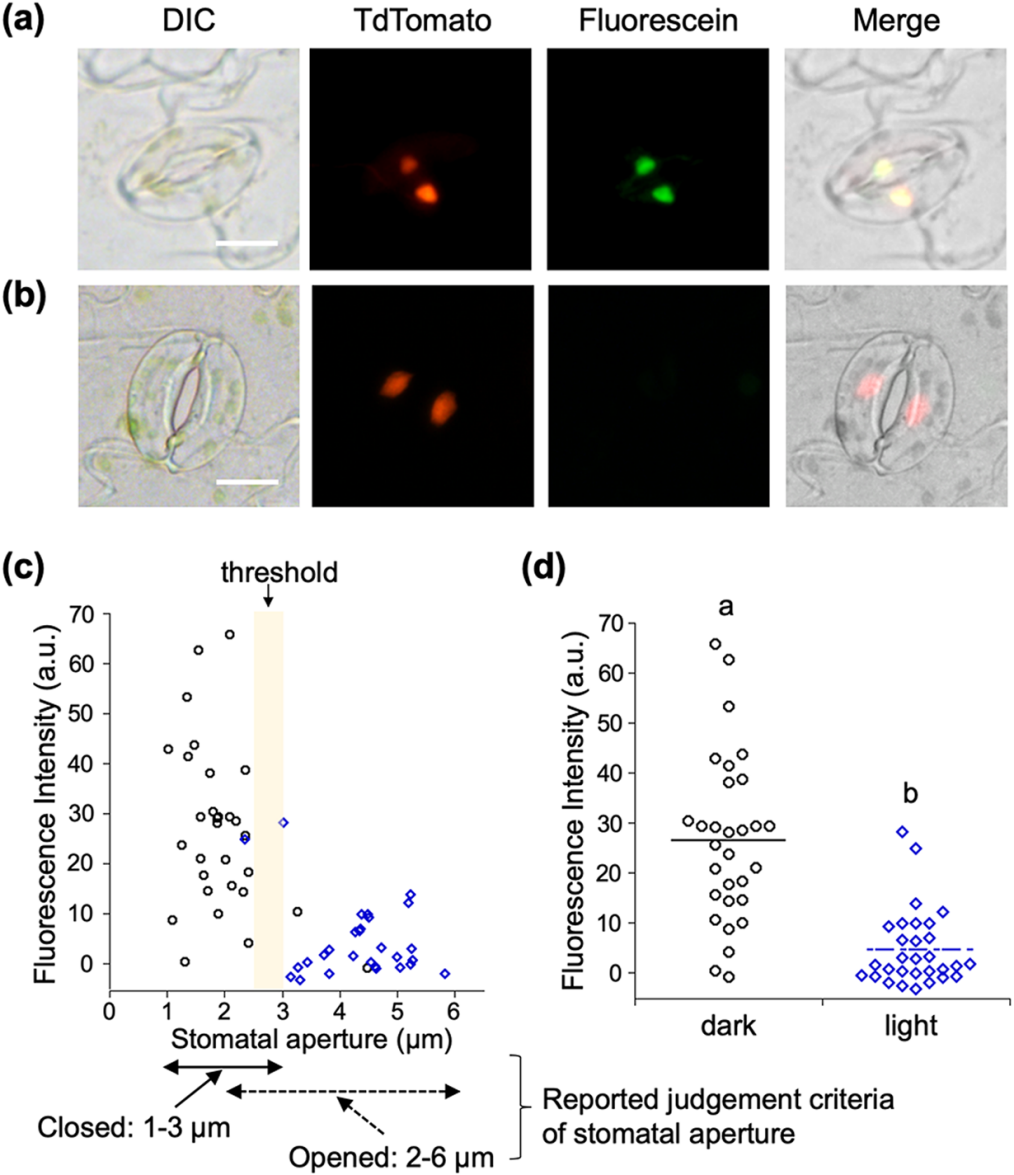
(**a**,**b**) Differential interference images (DIC) and fluorescent (tdTomato or Fluorescein) microscopic images of HoeAc_2_Fl-stained stomata of *P35S ::H2B-tdTomato*_*35S*_*::H2B-tdTomato* in the dark (**a**) or light (**b**) conditions; only the closed stomata were stained with HoeAc_2_Fl (the stomatal aperture was 2.15 µm), whereas opened stomata were not (the stomatal aperture was 4.70 µm) (see images in the fluorescein channel). The scale bars, 10 µm. (**c**) Relationship between stomatal apertures and fluorescent intensity of the nucleus of HoeAc_2_Fl-stained guard cells in the dark (black circle) or light condition (blue diamond). (**d**) Dotted plot of the fluorescence intensity of HoeAc_2_Fl-stained stomata in the dark (black circle) or light (blue diamond) conditions. Bars represent mean fluorescence intensity (*n* = 30). Significant differences were evaluated by one-way ANOVA/Tukey HSD post hoc test (*p* < 0.01).

**Figure 3:**
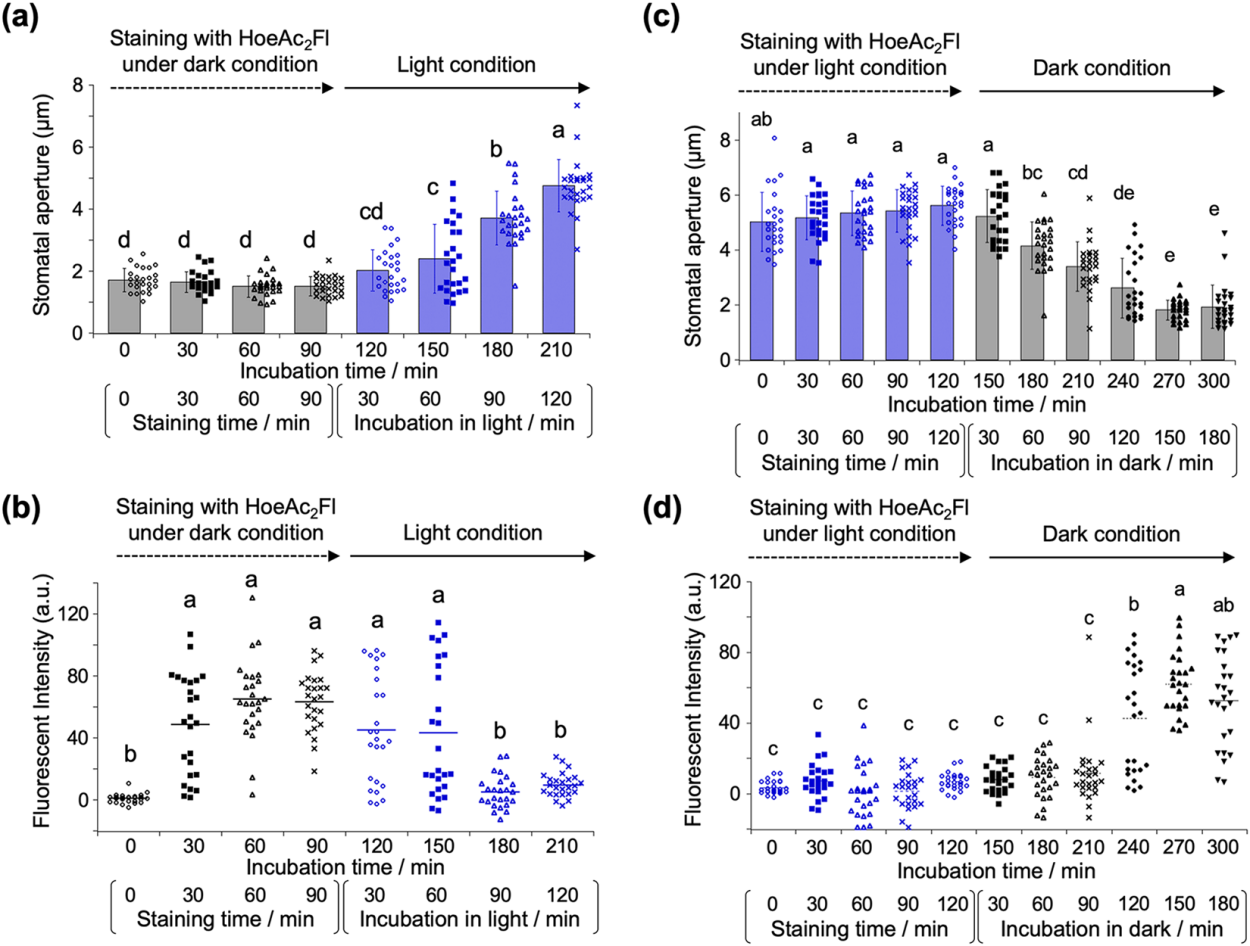
(**a**) Stomatal aperture of Col-0 during the staining process with HoeAc_2_Fl in the dark (0–90 min) and stomatal opening process in the light condition (total incubation time was 120-210 min, which is incubated for 30–120 min in the light condition). Error bars represent mean and SD (*n* = 25). (**b**) Dotted plot of the fluorescence intensity of staining process in the dark (0–90 min) and stomatal opening process in the light condition (total incubation time was 120–210 min, which is incubated for 30–120 min in the light condition) of HoeAc_2_Fl. Bars represent mean fluorescence intensity (*n* = 25). (**c**) Stomatal aperture of Col-0 during the staining process with HoeAc_2_Fl in the light (0–120 min) and stomatal closing process in the dark condition (total incubation time was 150–300 min, which is incubated for 30–180 min in the dark condition). Error bars represent mean and SD (*n* = 25). (**d**) Dotted plot of the fluorescence intensity of staining process in the light (0–120 min) and stomatal closing process in the dark condition (total incubation time was 150–300 min, which is incubated for 30–180 min in the dark condition) of HoeAc_2_Fl. Bars represent mean fluorescence intensity (*n* = 25). Significant differences were evaluated by one-way ANOVA/Tukey HSD post hoc test (*p* < 0.01).

**Figure 4:**
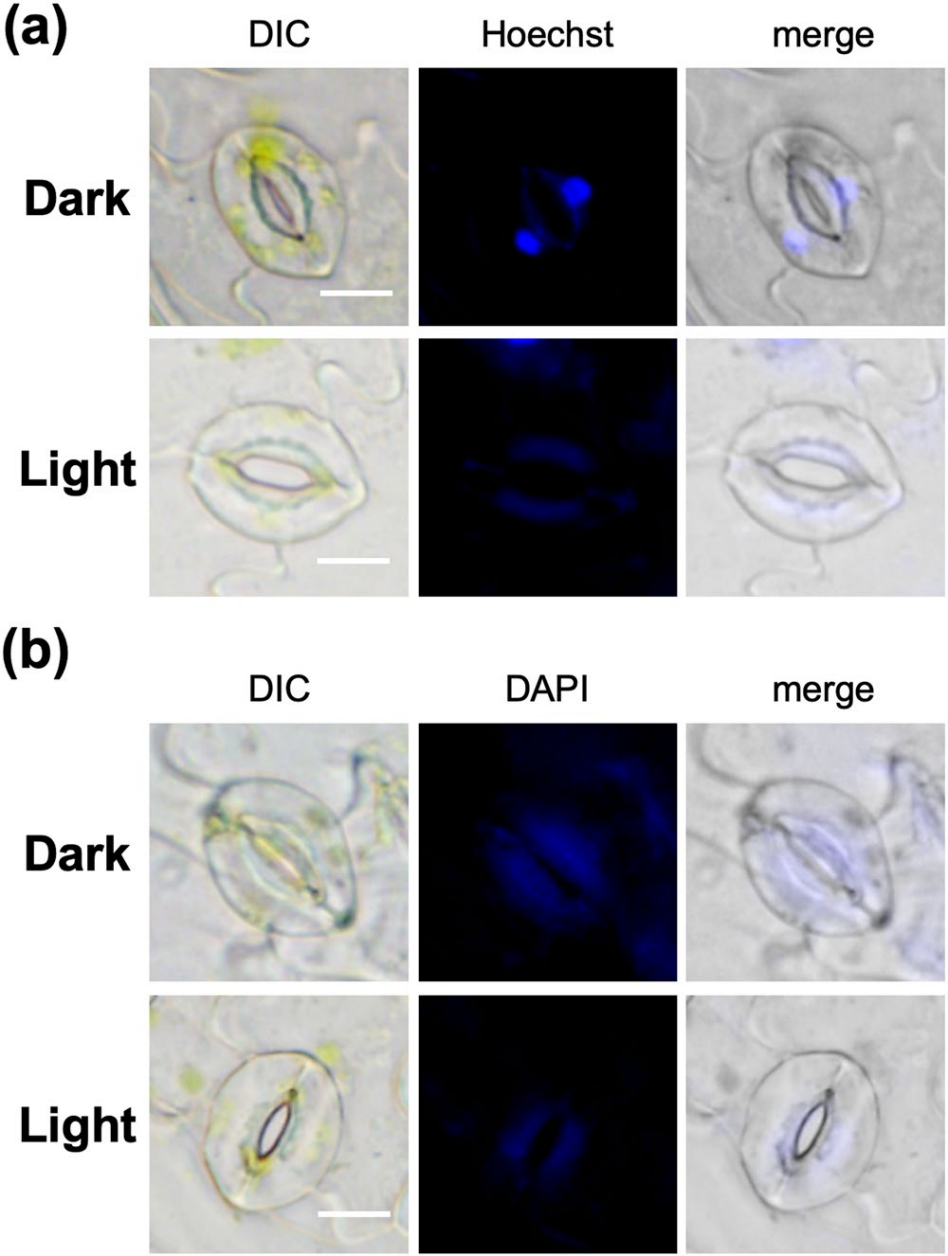
Fluorescent microscopic images of stomata of Col-0 stained by Hoechst 33342 (20 µg/mL, (**a**) or DAPI (20 µg/mL, (**b**) in the dark (top) or light (bottom) conditions. The scale bars, 10 µm.

Our studies commenced with the evaluation of the exact intracellular localization of HoeAc_2_Fl using the stomata of red fluorescent protein-fused histone protein-overexpressing plants (*P*_*35S*_*::H2B-tdTomato*), wherein the red fluorescence of H2B-tdTomato is localized in the nuclei^20^. After closure of the stomata by leaving the plant in the dark, the stomata were stained with HoeAc_2_Fl. The characteristic green fluorescence of HoeAc_2_Fl was observed from the nuclei of almost all of the closed stomata, co-localizing with H2B-tdTomato (Fig. 2a). In contrast, when the stomata were treated in the light, almost no fluorescence was observed from HoeAc_2_Fl-stained stomata (Fig. 2b). The exact correlation between fluorescence intensity and stomatal aperture showed that the visible fluorescence was only observed from stomata with an aperture of less than about 2.5–3 µm (Fig. 2c, three representative images of closed or open stomata are shown in Fig. S1, and fluorescence images of various stomata having different apertures are shown in Fig. S2). This threshold of the stomatal aperture is very close to the previously reported standard of aperture for determining opened/closed stomata, wherein stomata with an aperture of 1–3 µm are said to be closed; and those with an aperture of 2–6 µm are said to be open^5^. The mean fluorescence intensity of the nucleus in guard cells in the dark conditions was significantly higher than that in the light conditions (Fig. 2d), clearly demonstrating that HoeAc_2_Fl can only stain closed stomata. From the time course of the staining process with HoeAc_2_Fl, 60–90 min of incubation in the dark is enough to obtain the robust fluorescence intensity (Fig. 3a,b). After staining, the fluorescence intensity in nucleus was gradually decreased in the light condition according with gradual opening of stomata (Fig. 3a,b). In contrast, after incubation in the light condition, the fluorescence intensity increased in accordance with the stomatal closure under the dark. That is, the stomata gradually closed from 90 min (210 min in total, Fig. 3c) and completely closed around 150 min (270 min in total, Fig. 3c). Concurrently, the fluorescence intensity increased from 90 min (210 min in total, Fig. 3d) and reached plateau around 150 min (270 min in total, Fig. 3d). These results clearly demonstrated that the nuclear staining by HoeAc_2_Fl is reversible and depends on the stomatal dynamics. Similar results were obtained using Hoechst 33342, which was also found to stain closed but not open stomata, although some nonspecific staining was also observed at the edge of the stomata (Fig. 4a). This result strongly suggests that the unique localization property of HoeAc_2_Fl can be attributed to the Hoechst 33342 moiety. In contrast, almost no staining was observed by 4′,6-diamidino-2-phenylindole (DAPI)^21,22^, a conventional nucleus-staining fluorescent reagent^10^ (Fig. 4b). These results establish HoeAc_2_Fl as a practical chemical tool for the rapid identification of open/closed stomata; and its superior photochemical properties (such as longer excitation and emission wavelengths, and higher quantum yield) compared to Hoechst 33342 make it more useful.

Next, we sought to account for the selectivity of HoeAc_2_Fl for closed over open stomata. Our hypothesis was the efflux of HoeAc_2_Fl from open stomata precludes their staining. Accordingly, we carried out the staining experiment at a lower temperature, to suppress the transport activity. However, nuclear staining was unaffected, in spite of the significant decrease in the stomatal aperture under low temperature (Fig. S3). This result indicates that no transporter is involved in the efflux of HoeAc_2_Fl from the opened stomata. A mechanistic explanation for the selective nuclear localization of HoeAc_2_Fl is therefore unclear, and remains to be clarified.

**Figure 5:**
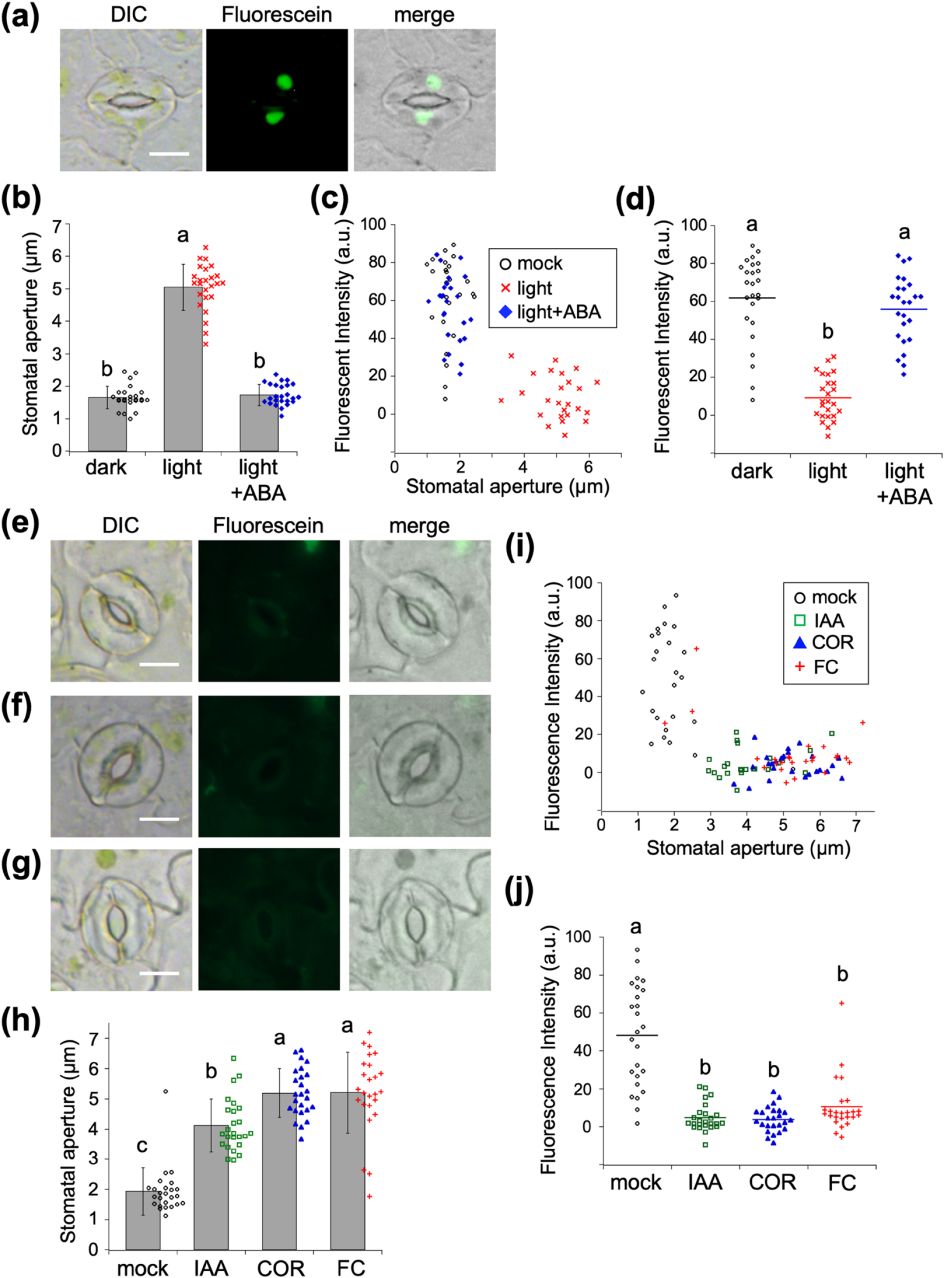
(**a**) Fluorescent microscopic images of HoeAc_2_Fl-stained stomata of Col-0 treated with ABA (10 µM) in the light. The scale bars, 10 µm. (**b**) Stomatal aperture of Col-0 treated without or with ABA (10 µM). Error bars represent mean and SD (*n* = 25). (**c**) Relationship between stomatal apertures and fluorescence intensity of the nucleus of HoeAc_2_Fl-stained guard cells in the mock condition (black circle), in the absence (red cross) or the presence of ABA (blue diamond). (**d**) Dotted plot of the fluorescence intensity of HoeAc_2_Fl-stained stomata in the mock condition (black circle), in the absence (black cross) or the presence of ABA (black diamond). (**e**–**g**) Fluorescent microscopic images of HoeAc_2_Fl-stained stomata of Col-0 treated with IAA (10 µM, **e**), COR (10 µM, **f**), or FC (10 µM, **g**) in the dark. The scale bars, 10 µm. (**h**) Stomatal aperture of Col-0 treated without or with various chemicals (IAA, COR, and FC, 10 µM). Error bars represent mean and SD (*n* = 25). Significant differences were evaluated by one-way ANOVA/Tukey HSD post hoc test (*p* < 0.01). (**i**) Relationship between stomatal apertures and fluorescence intensity of the nucleus of HoeAc_2_Fl-stained guard cells in the mock condition (black circle) or treated with IAA (green square), COR (blue triangle), or FC (red cross). (**j**) Dotted plot of the fluorescence intensity of HoeAc_2_Fl-stained stomata in the mock condition (black circle) or treated with IAA (green square), COR (blue triangle), or FC (red cross). Bars represent mean fluorescence intensity (*n* = 25). Sig*n*ificant differences were evaluated by one-way ANOVA/Tukey HSD post hoc test (*p* < 0.01).

We next used HoeAc_2_Fl to study chemically-triggered stomata dynamics^23^. Treatment of plants with abscisic acid (ABA) in the light has been previously reported to close stomata^24^. The peeled epidermis was stained by HoeAc_2_Fl followed by treatment of ABA in the light condition, fluorescence was observed from the nucleus of their guard cells (Fig. 5a–d). In contrast, treatment with auxin (IAA)^25^, coronatine (COR)^2,26,27^, and fusicoccin (FC)^28,29^ opened most of the stomata, and almost no fluorescence was observed in the nucleus of their guard cells (Fig. 5e–i). Similar to the light-triggered stomatal dynamics, the correlations between fluorescent intensity and stomatal aperture also demonstrated that fluorescence was only observed from closed stomata (Fig. 5i). Remarkable differences in the mean fluorescence intensities of ABA-treated stomata and IAA/COR/FC-treated stomata (Fig. 5d,j) were observed, confirming the applicability of HoeAc_2_Fl to the assessment of chemically induced stomatal dynamics. It is already well known that aperture lengths for open (2–6 µm) and closed (1–3 µm) stomata partly overlap in 2–3 µm^5^, and this marginal aperture length often cause difficulties to determine the results in stomatal bioassays. We examine the reliability of our method among this marginal region of aperture (2–3 µm). In the dose-dependent addition of COR, 0.3 µM of COR cause the stomatal aperture of the marginal length (2–3.5 µm) (Fig. S4a). Under the same condition, the mean fluorescent intensity of HoeAc_2_Fl was very close to that of open stomata treated by>1 µM of COR and significantly lower than that in the mock condition (Fig. S4b). Similar results were also obtained by the dose-dependency of FC (Fig. S5) and light intensity (Fig. S6). These results clearly demonstrated that our method judged that stomata of marginal aperture length belongs to the ‘open stomata’ and enabled clear decision of the results.

## Conclusion

HoeAc_2_Fl is proposed as a tool to easily and quickly assess whether plant stomata are open or closed based on its selectivity for the guard cells of closed stomata. The mechanistic basis for this selectivity is unknown. When the stomata were stained by HoeAc_2_Fl, the fluorescence was observed only from closed stomata. The clear threshold of the fluorescence provides objective criteria for the assessment of stomatal dynamics, although it is not quantitative. Instant determination of stomatal dynamics by measuring the fluorescence of HoeAc_2_Fl with objective analyses is expected to enable high-throughput screening of chemical libraries, which may lead to the discovery of novel chemical probes that can improve our understanding of plant responses to changes in their environments, and ultimately lead to improved crop production.

## Supplementary information

Supplementary Information.
