# Supplementary material for: Hoechst-tagged Fluorescein Diacetate for the Fluorescence Imaging-based Assessment of Stomatal Dynamics in Arabidopsis thaliana
Source: Sci Rep. 2020 Mar 24;10:5333. doi: 10.1038/s41598-020-62239-w (PMC7093514; doi:10.1038/s41598-020-62239-w)
Supplement: Supplementary file 1 — Supplementary Information. [file 41598_2020_62239_MOESM1_ESM.docx]

Supplementary Information for *Sci. Rep.*:

Yousuke Takaoka^1, 2^, Saki Miyagawa^3^, Akinobu Nakamura^4^, Syusuke Egoshi^1^, Shinya Tsukiji*^4, 5,6^, Minoru Ueda*^1,3^

^1^Department of Chemistry, Graduate School of Science, Tohoku University, Sendai 980-8578, Japan

^2^Precursory Research for Embryonic Science and Technology (PREST), Japan Science and Technology Agency, 5 Sanbancho, Chiyoda-ku, Tokyo 102-0075, Japan

^3^Department of Molecular and Chemical Life Sciences, Graduate School of Life Sciences, Tohoku University, Sendai 980-8578, Japan

^4^Department of Life Sciences and Applied Chemistry, Nagoya Institute of Technology, Gokiso-cho, Showa-ku, Nagoya 466-8555, Japan

^5^Department of Nanopharmaceutical Sciences, Nagoya Institute of Technology, Gokiso-cho, Showa-ku, Nagoya 466-8555, Japan

^6^Frontier Research Institute for Materials Science (FRIMS), Nagoya Institute of Technology, Gokiso-cho, Showa-ku, Nagoya 466-8555, Japan

*Correspondence: minoru.ueda.d2@tohoku.ac.jp, stsukiji@nitech.ac.jp

**Contents**

**Figure S1-S6.**

**Experimental section**


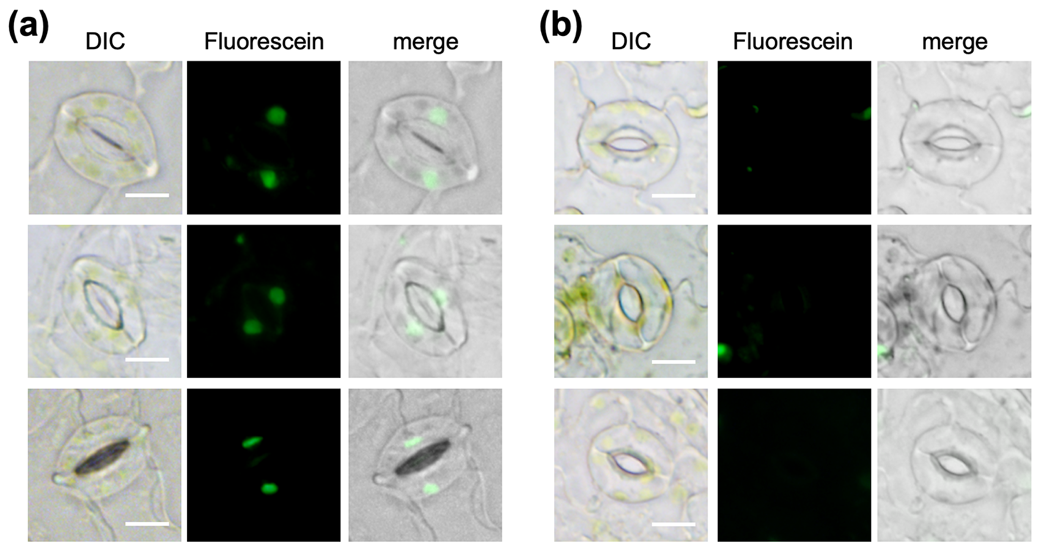


**Figure S1.** (a) Representative differential interference images (DIC) and fluorescent (fluorescein) microscopic images of HoeAc_2_Fl-stained stomata in the dark. (b) Representative differential interference images (DIC) and fluorescent (fluorescein) microscopic images of HoeAc_2_Fl-stained stomata in the light. The scale bars, 10 µm.


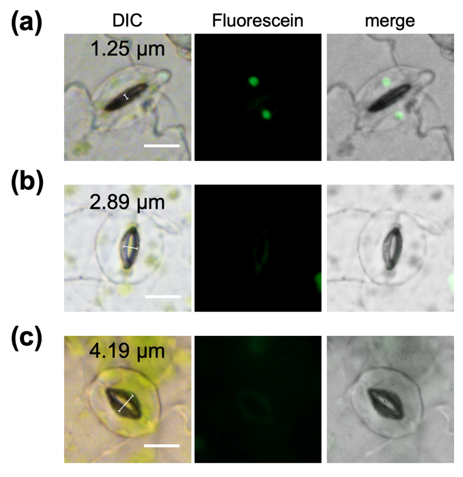


**Figure S2.** Differential interference images (DIC) and fluorescent (fluorescein) microscope images of HoeAc_2_Fl-stained stomata having various stomatal apertures (a: 1.25 µm, b: 2.89 µm, c: 4.19 µm). The scale bars, 10 µm.

**
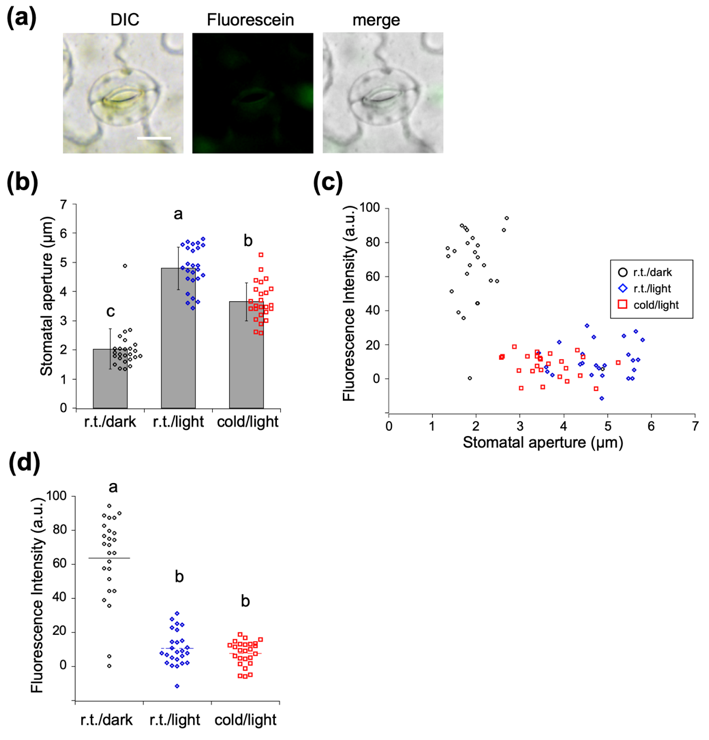
**

**Figure S3.** (a) Fluorescent microscope images of HoeAc_2_Fl-stained stomata of Col-0 in the light at 4 ºC. The scale bar, 10 µm. (b) Stomatal aperture of HoeAc_2_Fl-stained stomata of Col-0 in the dark or light at room temperature or 4ºC. Error bars represent the mean and SD (*n* = 25). Significant differences were evaluated by one-way ANOVA/Tukey HSD post hoc test (*p* < 0.01). (c) Relationship between stomatal apertures and fluorescence intensity of the nucleus of HoeAc_2_Fl-stained guard cells in the dark or light at room temperature or 4ºC. (d) Dotted plot of the fluorescence intensity of HoeAc_2_Fl-stained stomata in the dark or light conditions at room temperature or 4ºC. Bars represent mean fluorescence intensity (*n* = 25). Significant differences were evaluated by one-way ANOVA/Tukey HSD post hoc test (*p* < 0.01).


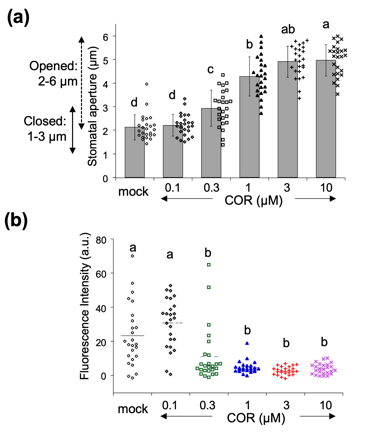


**Figure S4.** (a) Stomatal aperture of HoeAc_2_Fl-stained stomata of Col-0 treated with COR (0 – 10 µM) in the dark at room temperature. Error bars represent the mean and SD (n = 25). Significant differences were evaluated by one-way ANOVA/Tukey HSD post hoc test (p < 0.01). (b) Dotted plot of the fluorescence intensity of HoeAc_2_Fl-stained stomata treated with COR (0 – 10 µM) in the dark at room temperature. Bars represent mean fluorescence intensity (n = 25). Significant differences were evaluated by one-way ANOVA/Tukey HSD post hoc test (p < 0.01).


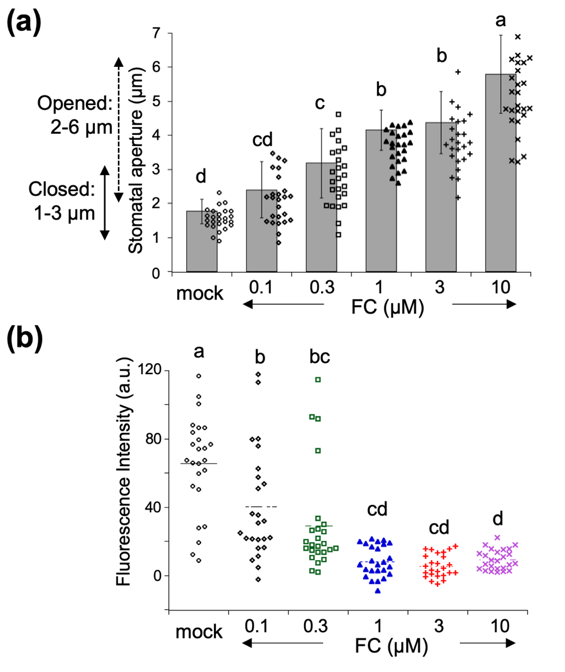


**Figure S5.** (a) Stomatal aperture of HoeAc_2_Fl-stained stomata of Col-0 treated with FC (0 – 10 µM) in the dark at room temperature. Error bars represent the mean and SD (n = 25). Significant differences were evaluated by one-way ANOVA/Tukey HSD post hoc test (p < 0.01). (b) Dotted plot of the fluorescence intensity of HoeAc_2_Fl-stained stomata treated with FC (0 – 10 µM) in the dark at room temperature. Bars represent mean fluorescence intensity (n = 25). Significant differences were evaluated by one-way ANOVA/Tukey HSD post hoc test (p < 0.01).


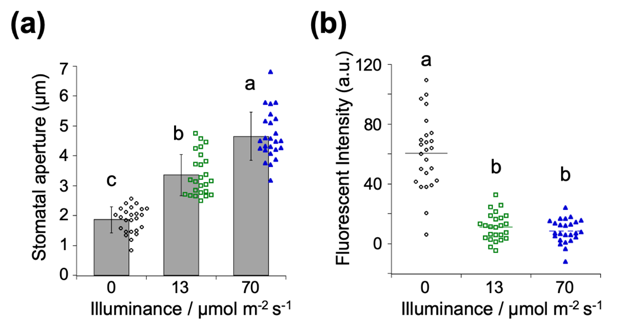


**Figure S6.** (a) Stomatal aperture of HoeAc_2_Fl-stained stomata of Col-0 incubated with different illuminance (0 – 70 µmol m^-2^ s^-1^) in the dark at room temperature. Error bars represent the mean and SD (n = 25). Significant differences were evaluated by one-way ANOVA/Tukey HSD post hoc test (p < 0.01). (b) Dotted plot of the fluorescence intensity of HoeAc_2_Fl-stained stomata incubated with different illuminance (0 – 70 µmol m^-2^ s^-1^) in the dark at room temperature. Bars represent mean fluorescence intensity (n = 25). Significant differences were evaluated by one-way ANOVA/Tukey HSD post hoc test (p < 0.01).

**Experimental Section**

**General Methods and Materials**

All chemical reagents were obtained from commercial suppliers (Kanto Chemical Co. Ltd., Wako Pure Chemical Industries Co. Ltd., Nacalai Tesque Co., Ltd., and Tokyo chemical industry Co., Ltd). *Arabidopsis thaliana* (ecotype Col-0 and *P_35S_::H2B-TdTomato* in Col-0 background) was grown under a 12 h light (70 µmol m^-2^ s^-1^; cool-white fluorescent light)/12 h-dark cycle at 22 °C on soil in a Biotron LPH-240SP growth chamber (Nippon Medical & Chemical Instruments Co. Ltd., Osaka, Japan).

**Staining and live imaging of peeled stomata**

The abaxial leaf epidermis of 5-week-old *Arabidopsis* plants was peeled and cut in squares approximately 2 mm × 2 mm in area. The epidermises were soaked in buffer (10 mM MES-KOH, 50 mM KCl, pH 6.2) containing HoeAc_2_Fl (20 µM) and incubated for 1.5 hours in the light or dark in the growth chamber. In the case of staining experiments with Hoechst 33342 or DAPI, the peeled epidermises were soaked in the same buffer containing each probe (20 µg/mL) and incubated for 10 min (for Hoechst 33342) or 1.5 hours (for DAPI) in the dark condition in the growth chamber.^1,2^ The images were taken on an IX71 microscope (Olympus Corp. Japan) equipped with a DP72 CCD camera and NUA filter (for Hoechst 33342), NIBA filter (for fluorescein) or WIGA filter (for TdTomato) (Olympus Corp). The length of stomatal apertures was measured using ImageJ 1.52a software (http://imagej.net/Welcome). The data were analyzed by one-way ANOVA/Tukey HSD post hoc test (*p* < 0.01). Statistical analysis was conducted using CoStat version 6.400 software (CoHort Software, http://www.cohort.com).

**Treatment of the chemicals for stomatal opening and closing**

The peeled epidermis was prepared as described above. Before the treatment of test compounds (coronatine and fusicoccin), the epidermises were soaked in the MES buffer containing HoeAc_2_Fl (20 µM) and closed under dark conditions at 22 ºC for 1.5 hours. Then, closed stomata were incubated with each compound (10 µM) in MES buffer at 22 ºC for 2 hours in the dark. The images were taken as described above.

**Treatment of IAA for stomatal opening**

The abaxial leaf epidermis of 5-week-old *Arabidopsis* plants was peeled and cut in squares approximately 2 mm × 2 mm in area. Then, the epidermises were soaked in 2 mL MES buffer (10 mM MES-KOH, 50 mM KCl, pH 6.2) in a Cell Culture Dish (35 mm×12 mm style, Nest Biotechnology Co., Ltd.). These epidermises were transferred 180 μL MES buffer containing HoeAc_2_Fl (20 µM, from 10 mM DMSO stock, 0.2 % DMSO contents) in a 48 well plate (sterilized, flat bottom 48 well plate for cell culture, Corning Inc.) and the stomata were closed under dark conditions in a Biotron LPH-240SP growth chamber at 22 ºC for 1.5 hours. Then, 20 μL of indole-3-acetic acid stock solution (100 µM in 20 % EtOH solution, Wako Pure Chemical Industries Co. Ltd.) were added (final 10 μM, 2% EtOH contents) and left to stand in growth chamber at 22 ºC for 2 hours in the dark. The images were taken as described above.

**Treatment of ABA for stomatal closing**

The peeled epidermis was prepared as described above. Before the treatment of ABA, the epidermises were soaked in the MES buffer containing HoeAc_2_Fl (20 µM) and closed under dark conditions at 22 ºC for 1.5 hours. Then, the epidermises were incubated with ABA (10 µM, Tokyo chemical industry Co., Ltd, from 100 µM 20 % EtOH stock, 2% EtOH contents) in MES buffer at 22 ºC for 2 hours in the light (70 µmol m^-1^ s^-1^). The images were taken as described above.

**Dose dependency of illuminance**

The peeled epidermis was prepared as described above. The epidermises were soaked in the MES buffer containing HoeAc_2_Fl (20 µM) and closed under dark conditions at 22 ºC for 1.5 hours, and then, rt at 22 ºC for 2 hours in the light conditions (70 or 13 µmol m^-2^ s^-1^, those were controlled by the settings of the growth chamber) or dark. The images were taken as described above.

**Time course of staining nucleus by HoeAc_2_Fl**

The peeled epidermis was prepared as described above. For the time course of the staining process in the dark and stomatal opening process in the light condition, the epidermises were soaked in the MES buffer containing HoeAc_2_Fl (20 µM) under the dark conditions at 22 ºC for 0, 30, 60 or 90 min incubation, and then transferred under light conditions (70 μmol m^-2^ s^-1^) for 30, 60, 90 or 120 min (total incubation time was 120, 150, 180, or 210 min, respectively). For the time course of the staining process in the light and stomatal closing process in the dark condition, the epidermises were soaked in the MES buffer containing HoeAc_2_Fl (20 µM) under the light conditions (70 μmol m^-2^ s^-1^) at 22 ºC for 0, 30, 60, 90 or 120 min incubation, and then transferred under dark conditions for 30, 60, 90, 120, 150 or 180 min (total incubation time was 150, 180, 210, 240, 270 or 300 min, respectively). The images were taken at each time period.

1 Vahisalu, T. *et al.* SLAC1 is required for plant guard cell S-type anion channel function in stomatal signalling. *Nature* **452**, 487-491 (2008).

2 Negi, J., Hashimoto-Sugimoto, M., Kusumi, K. & Iba, K. New approaches to the biology of stomatal guard cells. *Plant Cell Physiol* **55**, 241-250 (2014).
